# Role and Mechanisms of RAGE-Ligand Complexes and RAGE-Inhibitors in Cancer Progression

**DOI:** 10.3390/ijms21103613

**Published:** 2020-05-20

**Authors:** Ali H. El-Far, Grazyna Sroga, Soad K. Al Jaouni, Shaker A. Mousa

**Affiliations:** 1 Department of Biochemistry, Faculty of Veterinary Medicine, Damanhour University, Damanhour, Damanhour 22511, Egypt; ali.elfar@damanhour.edu.eg; 2 Rensselaer Polytechnic Institute, NY (RPI), Troy, NY 12180, USA; srogag@rpi.edu; 3 Department of Hematology/Pediatric Oncology, King Abdulaziz University, Yousef Abdulatif Jameel Scientific Chair of Prophetic Medicine Application, Faculty of Medicine, King Abdulaziz University, Jeddah 21589, Saudi Arabia; saljaouni@kau.edu.sa; 4 The Pharmaceutical Research Institute, Albany College of Pharmacy and Health Sciences, Rensselaer, NY 12144, USA

**Keywords:** RAGE-ligands, AGEs, HMGB1, S100s, RAGE-inhibitors, carcinogenesis

## Abstract

Interactions of the receptor for advanced glycation end product (RAGE) and its ligands in the context of their role in diabetes mellitus, inflammation, and carcinogenesis have been extensively investigated. This review focuses on the role of RAGE-ligands and anti-RAGE drugs capable of controlling cancer progression. Different studies have demonstrated interaction of RAGE with a diverse range of acidic (negatively charged) ligands such as advanced glycation end products (AGEs), high-mobility group box1 (HMGB1), and S100s, and their importance to cancer progression. Some RAGE-ligands displayed effects on anti- and pro-apoptotic proteins through upregulation of the phosphatidylinositide 3-kinase (PI3K)/protein kinase B (Akt)/mammalian target of rapamycin (mTOR), mitogen-activated protein kinases (MAPKs), matrix metalloproteinases (MMPs), vascular endothelial growth factor (VEGF), and nuclear factor kappa B (NF-***κ***B) pathways, while downregulating p53 in cancer progression. In addition, RAGE may undergo ligand-driven multimodal dimerization or oligomerization mediated through self-association of some of its subunits. We conclude our review by proposing possible future lines of study that could result in control of cancer progression through RAGE inhibition.

## 1. Introduction 

The receptor for advanced glycation end products (RAGE) is involved in various inflammatory and immune responses [1]. Structurally, RAGE is a 45-kDa protein that is highly expressed during embryonic development, especially in the brain, and then its expression decreases in adult tissues [2]. RAGE has been recognized in several diseases with different isoforms including the full-length RAGE, dominant negative RAGE (DN-RAGE), N-truncated RAGE (N-RAGE), and C-truncated secretory/soluble RAGE (sRAGE) [3,4]. RAGE is overexpressed on cell surface membrane of activated immune, vascular, and cancer cells [5]. Binding of advanced glycation end products (AGEs), S100 proteins, high-mobility group box1 (HMGB1), amyloid β-peptide, lipopolysaccharides, β-sheet fibrils, advanced oxidation protein products, Mac-1, or phosphatidyl serine with RAGE directs the cancer cells toward survival and proliferation [6,7,8]. Interaction of RAGE with these molecules activates cell signaling pathways such as mitogen-activated protein kinase (MAPK) and nuclear factor kappa B (NF-***κ***B), which induce cellular propagation [9,10]. As a result, ligands-induced RAGE signaling increases the malignant progression of glioma, bladder, melanoma, liver, pancreatic, prostate, colorectal, gastric, and lung cancers [11,12,13,14].

Apoptosis is a programmed cell death, which plays a significant role in tumor invasiveness and metastasis. Any defects in apoptosis play a pivotal role in initiation and progression of carcinogenesis [15]. Hence, cancer cell progression is induced by downregulation of the intrinsic and extrinsic pathways of apoptosis [16,17]. RAGE interferes with apoptosis through a p53-dependent mitochondrial pathway. Furthermore, RAGE controls autophagy through decreased phosphorylation of mammalian target of rapamycin (mTOR), the anti-apoptotic protein, and increased Beclin-1/VPS34 autophagosome formation [18]. 

In this review, we will discuss the new studies concerning ligands and inhibitors of RAGE and their roles in cancer progression. We recommend some future studies that could help to delineate the mechanism by which RAGE-ligands could induce cancer progression. We also provide a guide for targeted inhibition of RAGE by its ligands, which may serve as novel targets to improve current cancer therapies. 

## 2. Mechanisms of AGEs-RAGE Axis Cancer Progression

**Figure 1:**
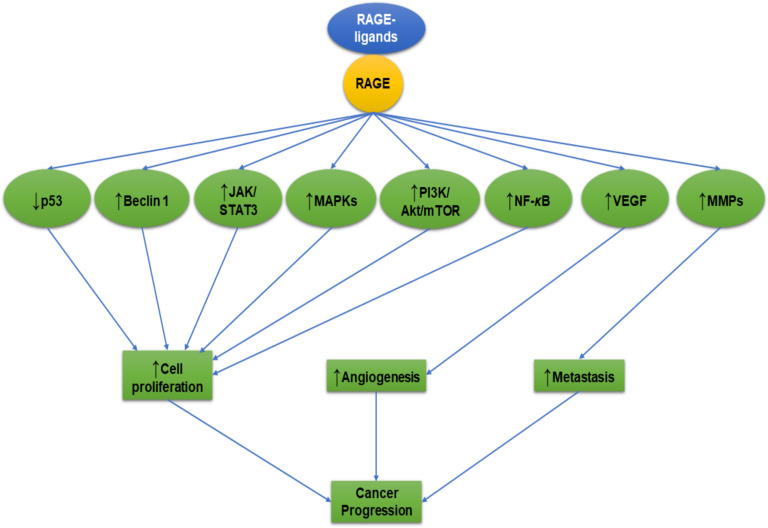
The effects of receptor for advanced glycation end product (RAGE)-ligands on cancer progression. The interaction between RAGE-ligands/RAGE results in upregulation of the phosphatidylinositide 3-kinase (PI3K)/protein kinase B (Akt)/mammalian target of rapamycin (mTOR), mitogen-activated protein kinases (MAPKs), matrix metalloproteinases (MMPs), vascular endothelial growth factor (VEGF), and nuclear factor kappa B (NF-***κ***B) pathways and downregulation of p53. These pathways play an important role in controlling the tumor cells’ proliferation, angiogenesis, and invasion. The arrow pointing up (↑) means increase or upregulation. The arrow pointing down (↓) means decrease or downregulation.

AGEs are formed through a nonenzymatic process also known as Maillard reaction. This reaction is initiated between the free amino group of a protein and a carbonyl group from a reducing sugar to produce AGEs [19] followed by a series of cascade reactions that include dehydration, oxidation, condensation, and cyclization [20]. The formed AGEs enhance cancer cell migration through the expression of vascular endothelial growth factor (VEGF), NF-***κ***B, and extracellular signal-regulated kinase (ERK) pathways [13,21]. AGEs-RAGE axis has induced cancer cell progression in colon, breast, oral, prostate, and neuroblastoma cell lines (Table 1), and AGEs- RAGE axis can enhance cancer progression through regulation of the pathways and molecules described below and illustrated in Figure 1 [21,22,23,24,25,26,27].

### 2.1. ChREBP

The AGEs studies presented in Table 1 indicate that colorectal and hepatocellular carcinoma (HepG2) cells are the ones most affected by AGEs through upregulation of carbohydrate response element binding protein (ChREBP) [28]. ChREBP binds to the carbohydrate response element in the promoter of pyruvate kinase, the enzyme that is involved in the processes of glycolysis, lipogenesis, and gluconeogenesis [29]. This pivotal role of ChREBP in cancer progression might be due to the enhancement of anaerobic glucose metabolism and suppression of p53 that favors cancer growth [30].

### 2.2. JAK/STAT3

Janus kinase (JAK)/signal transducer and activator of transcription (STAT) signaling plays an important role in a variety of diseases, including cancer [31]. JAK/STAT can facilitate the transcription of genes involved in cancer cell proliferation [32]. The AGEs-RAGE axis-induced upregulation of STAT3 was observed in erythroleukemia [33] and breast adenocarcinoma [25].

### 2.3. MAPK and MMPs

It was also established that after binding to RAGE, AGEs upregulated some MAPK pathways and related MAPK family members such as ERK, specificity protein 1 (Sp1), matrix metalloproteinase 2 (MMP2), MMP9, and p38 [13,25,27,34,35,36,37]. For example, after binding to RAGE, AGEs upregulated Sp1 expression via activating ERK pathway [34]. Notably, Sp1 can regulate many cellular molecules including MMP2, which can degrade type IV collagen to facilitate the metastasis of cancer cells [38], including human oral cancer (SAS) [39], gastric cancer cell line SGC7901 [34], and human colorectal cancer samples [35] via AGEs-RAGE axis. Moreover, another matrix metalloproteinase member, MMP9, which is upregulated by ERK after activation of AGEs-RAGE, is strongly associated with progression and metastatic breast and oral cancers [37,39].

### 2.4. Nrf-2

Nuclear factor (erythroid-derived 2)-like 2 (Nrf-2): Keap1 complex could lead to cancer cell proliferation and metastasis through regulation of p53 apoptotic pathway [23,40]. Activated AGEs-RAGE axis in oral cancer (SAS) cell line led to downregulation of Nrf-2 that consequently facilitated the spread of cancer cells [23]. The authors postulated that downregulation of Nrf-2 is associated with the downregulation of p53 of oral cancer cells via heme oxygenase (HO-1). This finding is in accordance with the study of Lee et al. [41] who stated that downregulation of Nrf-2 led to downregulation of HO-1, consequently downregulating p53, and hindering apoptosis of oral cancer cells—thus favoring their progression.

### 2.5. PI3K/Akt

A number of different studies demonstrated that induction of AGEs led to upregulation of phosphatidylinositide 3-kinase (PI3K) [22,33], protein kinase B (Akt) [22], NF-***κ***B [13,42,43,44], and VEGF [21,24] and downregulation of p53 [23] in various cancer cell types (Table 1). Based on these studies, we propose that activation of the AGEs-RAGE axis leads to upregulation of PI3K and subsequent phosphorylation of Akt. The aforementioned processes were accompanied by phosphorylation of Akt and enhanced the expressions of NF-***κ***B, VEGF, and mouse double minute 2 homolog (MDM2). These three molecules protected the cancer cells from apoptosis. Overexpressed NF-***κ***B resulted in increased cancer cells’ proliferation and protected them from apoptosis. Upregulation of NF-***κ***B abrogated the activities of the caspases with cancer proliferation [45]. Moreover, the upregulated VEGF is known to be the key mediator of angiogenesis that forms the new blood vessels for nutrition and propagation of cancer cells [46]. Finally, phospho-Akt upregulated the MDM2 protein that consequently downregulated p53 tumor suppressor gene, leading to the termination of both intrinsic and extrinsic apoptotic pathways and favoring cancer cell proliferation.

**Table 1:** Role of the advanced glycation end products (AGEs)-receptor for advanced glycation end product (RAGE) axis in cancer progression.

Cell Lines	Study Type/Samples	Mechanisms of Action	References
Human colon adenocarcinoma (Caco-2)	In vitro	↑ERK1/2, ↑MAPK	[27]
Human colon carcinoma (Colo320 and WiDr)	In vitro	↑NF-***κ***B, ↑ERK	[13]
Human erythroleukemia (HEL)	In vitro	↑MAPK, ↑PI3K, ↑JAK/STAT	[33]
Human breast cancer (MCF-7)	In vitro	↑VEGF	[24]
Human breast tumor samples	In vivo	↑NF-***κ***B	[42]
Human hepatocellular carcinoma (Hep3B and HepG2)	In vitro	↑VEGF	[21]
Glioma (C6)	In vitro	↑NF-***κ***B	[43]
Human colorectal carcinoma (HCT116)	In vitro	↑ChREBP	[28]
Human hepatocellular carcinoma (HepG2)	In vitro	↑ChREBP	[28]
Human oral cancer (SAS)	In vitro	↑ERK, ↑MMP2, ↑MMP9	[39]
Human prostate cancer (PC-3)	In vitro	↑PI3K/Akt	[22]
Human breast cancer (MDA-MB-231)	In vitro	↑ERK1/2, ↑STAT3, ↑p38, ↑MAPK, ↑MMP-9	[25]
Human gastric cancer (SGC7901) and human gastric tumors samples	In vitro/ In vivo	↑ERK/Sp1/MMP2	[34]
Rat pheochromocytoma (PC12)	In vitro	↑NF-***κ***B	[44]
Human breast cancer (MCF-7)	In vitro	↑MMP9, ↑ERK1/2	[37]
Human oral cancer (SAS)	In vitro	↓Nrf-2, ↓p53	[23]
Colorectal cancer (human samples)	In vivo	↑ERK/SP1/MMP2	[35]

**Akt**; protein kinase B, **ERK**; extracellular signal-regulated kinase, **JAK**; Janus kinase, **MAPK**; mitogen-activated protein kinase, **MMPs**; matrix metalloproteinases, **NF-***κ***B**; nuclear factor kappa B, **Nrf-2**; Nuclear factor (erythroid-derived 2)-like 2, **PI3K**; phosphatidylinositide 3-kinase, **STAT3**; signal transducer and activator of transcription 3.The arrow pointing up (↑) means increase or upregulation.The arrow pointing down (↓) means decrease or downregulation.

## 3. Mechanisms of HMGB1-RAGE Axis in Cancer Progression

High-mobility group box1 (HMGB1) is a crucial factor in the development and progression of breast, colon, gastrointestinal, and liver cancers [12,47,48,49]. Binding of HMGB1 to RAGE has been associated with tumor cell survival, progression, and metastasis [50]. HMGB1 protects cells from apoptosis because it affects telomere stability and stimulates certain cellular proteins involved in the proliferation of cancer cells [51]. The effects of HMGB1 on RAGE and subsequent upregulation of certain anti-apoptotic molecules and downregulation of pro-apoptotic molecules in these studies are presented in Table 2. The mechanisms of their action are discussed next.

### 3.1. Beclin-1

Beclin-1 has been associated with cancer cell autophagy. However, Rohatgi and Shaw have determined that Beclin-1 induced tumor progression by regulation of growth factor receptor signaling in an autophagy-independent manner [52]. Beclin-1 regulated the epidermal growth factor and insulin-like growth factor-1, leading to activation of Akt and ERK in breast cancer cells [53]. In the same way, Beclin-1 was upregulated by AGEs and enhanced the progression and aggressiveness of human pancreatic cancer (Panc2.03) cell lines [54].

### 3.2. MAPK

Numerous studies have recognized the upregulation of the ERK/MAPK pathway by HMGB1 in colon carcinoma [13,55], renal cell carcinoma [56], liver carcinogenesis in mice [57], gastric carcinoma [58], hepatocellular carcinoma [59], breast adenocarcinoma (MCF-7) [55], and alveolar basal epithelial cells adenocarcinoma (A549) cells [55]. Upregulation of the ERK/MAPK pathway induced the survival and proliferation of those cells. Activation of ERK enhanced the expression of c-Myc that downregulated p21 and enhanced the cell cycle members cyclin-dependent kinase-2 (CDK-2) and cyclin D1 in hepatocellular carcinoma [59]. In addition, ERK upregulated MMP2 and MMP9, which led to cell proliferation and metastasis [60]. Other MAPK members have been upregulated by HMGB1 including cell division control protein 42 homolog (Cdc42)/Ras-related C3 botulinum toxin substrate 1 (Rac1)/mitogen-activated protein kinase kinase 6 (MKK6)/p38 that led to the proliferation of human rhabdomyosarcoma [61] and hepatocellular carcinoma [59]. Additionally, another member of MAPKs, stress-activated protein kinases (SAPK)/ c-Jun N-terminal kinase (JNK), has been shown to be upregulated due to the HMGB1-RAGE activation in glioma, murine Lewis lung carcinoma, and hepatocellular carcinoma (HCC) [59,62].

### 3.3. MicroRNA

MicroRNAs (miRNAs or miR) are small noncoding RNAs that participate in the regulation of diverse cell functions [63]. The abnormally regulated miRNAs have been shown to affect cancer proliferation, invasion, and metastasis [64]. HMGB1-RAGE axis upregulated miR-221/222 in thyroid carcinoma [65,66]. The re-regulated expression of miR-221 and miR-222 in thyroid carcinoma decreased p27(Kip1) protein levels, a major cell cycle regulator, which contributed to the advance of thyroid carcinoma in the S-phase [67]. In addition, miR-155-5p was transferred to colorectal cancer cells by macrophage-derived exosomes and downregulated expression of BRG1, the key factor promoting the colorectal cancer metastasis [68]. Therefore, upregulation of these miRNAs with HMGB1-RAGE helps cancer progression.

### 3.4. MMPs

MMPs are a group of proteolytic enzymes that degrade multiple extracellular matrix components to promote tumor invasion, angiogenesis and metastasis, and therefore they represent ideal pharmacologic targets for cancer therapy [69]. HMGB1 promoted the upregulation of MMPs (MMP1, MMP2, MMP3, MMP7, MMP9, and MMP10). Upregulation of MMPs with HMGB1 has been associated with cancer cell proliferation of colon [13,70], pancreatic [71], pulmonary [60], hepatocarcinoma [72], and hepatocellular carcinoma cell lines [59]. The upregulated MMPs are capable of degrading the components of the extracellular matrix and facilitating tumor progression [73].

### 3.5. NF-κB/Snail

HMGB1 upregulated NF-***κ***B expression in human pancreatic (BxPC-3) [74], hepatocarcinoma (HUH 7 and H22) [14,72], fibrosarcoma (HT1080) [11,75], non-small cell lung cancer (NSCLC) [76], HCC [77], and breast adenocarcinoma (MCF-7) cells [78]. Overexpressed NF-***κ***B upregulated Snail, which subsequently upregulated N-cadherin [79] and vimentin [80]. N-cadherin and vimentin favor the epithelial-mesenchymal cells transition and cancer cell migration. In addition, Snail-induced downregulation of E-cadherin [79] and phosphatase and tensin homolog (PTEN) resulted in cell proliferation and metastasis [65,66].

### 3.6. PI3K/Akt

HMGB1 upregulated PI3K/Akt, which subsequently regulated various cellular proteins that induced cell proliferation and decreased apoptosis of cancer cells [55,81,82]. One of them is VEGF that overexpressed due to the increased level of HMGB1 in human oral squamous cell carcinoma (OSCC) [83,84] and bladder carcinoma [85], facilitating angiogenesis of cancer and its proliferation. In the same context, PI3K/Akt upregulation led to downregulation of pro-apoptotic molecules such as Bcl-2-associated X protein (Bax) and caspase-3 in human nasopharyngeal carcinoma (HONE-1) [86] and lung cancer cells (Lewis cells) [82].

**Table 2:** Role of the high-mobility group box1 (HMGB1-receptor for advanced glycation end product (RAGE) axis in cancer progression.

Cell Lines/Samples	Study Type	Mechanisms of Action	References
Glioma (C6)	In vitro	↑ERK1/2, ↑p38, ↑SAPK/JNK	[62]
Murine Lewis lung carcinoma	In vitro	↑ERK1/2, ↑p38, ↑SAPK/JNK	[62]
Mouse neuroblastoma (Neuro2a)	In vitro	↑Bcl-2	[87]
Human neuroblastoma (SH-SY5Y)	In vitro	↑Bcl-2	[87]
Human colon carcinoma (Colo320 and WiDr)	In vitro	↑ERK1/2, ↑Rac1, ↑Akt, ↑MMP9	[13]
Human pancreatic carcinoma (PANC-1 and MIA PaCa-2)	In vitro	↑MMP9	[71]
Human rhabdomyosarcoma (TE671)	In vitro	↑Cdc42-Rac1-MKK6-p38	[61]
Human oral squamous cell carcinoma (OSCC)	In vitro	↑VEGF	[83]
Human pancreatic adenocarcinoma (Panc 2.03)	In vitro	↑Beclin-1	[54]
Human oral squamous cell carcinoma (OSCC)	In vitro	↑VEGF	[84]
Human pancreatic cancer (BxPC-3)	In vitro	↑NF-***κ***B	[74]
Human esophageal squamous cell carcinoma (KYSE-150)	In vitro	↑VEGF-C	[88]
Human renal cell carcinoma (CCRCC)	In vitro	↑ERK1/2	[56]
Human thyroid carcinoma (BC PAP)	In vitro	↑miR-221/222, ↓PTEN	[66]
Human chondrosarcoma (JJ012)	In vitro	↑PI3K/Akt/c-Jun/AP-1, ↑α5β1 integrin	[81]
Human lung cancer (95D)	In vitro	↑MMP2, ↑MMP9, ↑CDK-2	[60]
Human hepatocarcinoma (HUH 7)	In vitro	↑NF-***κ***B, ↑p65	[14]
Human hepatocarcinoma (H22)	In vitro	↑NF-***κ***B, ↑MMP9	[72]
Liver carcinogenesis in mice	In vivo	↑ERK1/2, ↑Cyclin D1	[57]
Human fibrosarcoma (HT1080)	In vitro	↑NF-***κ***B	[75]
Mouse melanoma (B16-F10)	In vitro	↑STAT3	[89]
Human non-small cell lung cancer (NSCLC)	In vitro	↑JNK, ↑NF-***κ***B	[76]
Pancreatic tumor (human Panc2.03, human Panc3.27, mouse Panc02)	In vitro	↑ATP	[90]
Human hepatocellular carcinoma (HCC)	In vitro	↑NF-***к***B	[77]
Murine lung cancer (Lewis cells)	In vitro	↑PI3K/Akt, ↑ERK1/2, ↑Bcl-2, ↓Bax	[82]
Human breast cancer (MCF-7)	In vitro	↑NF-***κ***B	[78]
Human bladder carcinoma (5637, BIU-87, T24, and SV-HUC-1)	In vitro	↑NF-***κ***B, ↑VEGF	[85]
Human thyroid carcinoma (BC PAP)	In vitro	↑miR-221/222, ↓PTEN	[65]
Human nasopharyngeal carcinoma (HONE-1)	In vitro	↑Bcl-2, ↑p-ERK1/2, ↓caspase-3, ↓Bax	[86]
Murine Lung cancer (Lewis cells)	In vitro	↑ERK1/2	[91]
Human gastric carcinoma (BGC-823, SGC-7901, MKN-28, and MKN-45)	In vitro	↑ERK1/2	[58]
Human colorectal carcinoma (HCT116 and LoVo)	In vitro	↑-Snail/NF-***κ***B, ↑MMP7	[70]
Human hepatocellular carcinoma (HCC)	In vitro	↑MMP2, ↑ERK1/2, ↑p38, ↑SAPK/JNK, ↑MEK1/2, ↑SEK1, ↑c-Jun, ↑c-Myc, ↓p21	[59]
Human colorectal adenocarcinoma (HT-29)	In vitro	↑MAPK, ↑PI3K	[55]
Human breast cancer (MCF-7)	In vitro	↑MAPK, ↑PI3K	[55]
Human adenocarcinomic human alveolar basal epithelial (A549)	In vitro	↑MAPK, ↑PI3K	[55]
Human hypopharyngeal carcinoma (FaDu)	In vitro	↑Vimentin, ↑Snail	[80]
Cervical carcinomas (human specimens and HeLa cells)	In vivo	↑NF-***κ***B, ↑N-cadherin↓E-cadherin	[79]
Human colorectal carcinoma (LoVo)	In vitro	↑NF-***κ***B	[92]
Human prostate cancer (PC-3)	In vitro	↑NF-***κ***B, ↑MMP1, ↑MMP3, ↑MMP10	[93]

**Akt**; protein kinase B, **AP-1**; activator protein 1, **ATP**; adenosine triphosphate, **Bax**; Bcl-2-associated X protein, **Bcl-2**; B-cell lymphoma 2, **Cdc42**; cell division control protein 42 homolog, **CDK-2**; cyclin-dependent kinase-2, **ERK**; extracellular signal-regulated kinase, **JNK**; c-Jun N-terminal kinase, **MKK6**; mitogen-activated protein kinase kinase 6, **MMPs**; matrix metalloproteinases, **NF-***κ***B**; nuclear factor kappa B, **PI3K**; phosphatidylinositide 3-kinase, **PTEN**; phosphatase and tensin homolog, **Rac1**; Ras-related C3 botulinum toxin substrate 1, **SAPK**; stress-activated protein kinases, **STAT3**; signal transducer and activator of transcription 3, **VEGF**; vascular endothelial growth factor, **VEGF-C**; vascular endothelial growth factor C. The arrow pointing up (↑) means increase or upregulation. The arrow pointing down (↓) means decrease or downregulation.

## 4. Mechanisms of S100 Family-RAGE Axis in Cancer Progression

S100 proteins are involved in many aspects of regulation of proliferation, differentiation, apoptosis, and inflammation [94]. Cancers exhibit a distinctive S100 protein as determined in human osteosarcoma, melanoma, pancreatic carcinoma, colorectal carcinoma, and breast adenocarcinoma [95,96,97,98]. The data in Table 3 show the possible mechanisms by which S100 proteins could induce cancer progression associated with the RAGE signaling pathway. These mechanisms are discussed below.

### 4.1. Angiogenesis 

Angiogenesis is defined as the formation of new blood vessels from preexisting vessels and has been characterized as an essential process for cancer cell proliferation and viability because it supplies the cancer cells with nutrients and oxygen [99]. Notably, VEGF is a protein involved in both vasculogenesis and angiogenesis leading to growth and metastasis of solid cancers [100]. Therefore, targeting of VEGF/vascular endothelial growth factor (VEGFR) signaling is a powerful approach to restrict cancer progression [99]. Some studies listed in Table 3 revealed downregulation of VEGF expression in response to S100s-RAGE binding in pancreatic, colorectal, and breast cancers [101,102,103].

### 4.2. MAPK

S100s members upregulated MAPK family members either ERK, Cdc42/p38, or SAPK/ JNK pathways that led to the progression of colorectal (HCT116, MC38, SW620, and DLD-1), thyroid (human specimens), nasopharyngeal (human specimens), breast (human specimens), and prostate (LNCaP and PC-3) cancers [95,104,105,106,107,108,109]. These resulted in cancer proliferation and progression.

### 4.3. MMPs

Molecular-based assessments of breast cancers have revealed expression of S100s, including S100A2, S100A4, and S100A7, with different expression levels during breast tumorigenesis and progression [110]. Nasser et al. [108] observed a relation between S100A7 and cancer progression of aggressive triple-negative breast cancer of human specimens and found upregulation of MMP9 due to S100A7. Moreover, MMP9 expression was increased in pancreatic carcinoma (BxPC3) [111] and nasopharyngeal carcinoma (C666-1) along with MMP2 [112] that was triggered by S100P. These results encourage researcher to control breast, pancreatic, and nasopharyngeal cancers through blockage of S100A7 and S100P-RAGE.

### 4.4. NF-κB

Activation of RAGE by S100A4, S100A7, S100A8, S100A9, S100A14, S100B, and S100P was associated with upregulation of NF-***κ***B that induced progression of different types of cancer including pancreatic, melanoma, breast, prostate, colon, neuroblastoma, and esophageal [74,97,106,108,113,114,115]. S100A4 upregulated E-cadherin [101,116] that was regulated by NF-***κ***B/Snail and led to cancer metastasis. Thus, to understand the effects of cancer progression due to induction of the S100s family members through the NF-***κ***B pathway, it is essential to study their effects on the inhibitors of apoptosis members (IAPs), B-cell lymphoma (Bcl-2), and Snail.

### 4.5. p53

Because of stimulation of RAGE by the S100B in melanoma, lung, breast, colorectal, and ovarian cancers [117,118,119,120] by p53 downregulation that inhibited the intrinsic and extrinsic apoptotic pathways leading to cancer progression. In the aforementioned studies, the authors investigated the effects of S100B on p53, but they did not study the effect on the other members of intrinsic and extrinsic apoptotic pathways such as caspase-3, -8, and -9. Therefore, we propose that it is important to study the effect of S100B on intrinsic and extrinsic apoptotic pathway molecules. In addition, p53 regulated the cell cycle via p21. Recent data revealed that S100A16 caused significant decreases in p21 and p27 expressions in human prostate cancer and this promoted the cycle division of cancer cells [121]. 

### 4.6. PI3K/Akt/mTOR

The PI3K/Akt/mTOR pathway exists as one of the most attractive targets to block cancer progression [122]. This pathway has been upregulated by S100A4 in colorectal [101], S100A16 in prostate [121], and S100B in neuroblastoma cancers [113] with growth promotion caused by the anti-apoptosis and cell proliferation induced by the PI3K/Akt/mTOR pathway.

### 4.7. STAT3

Diverse studies have demonstrated constitutive activation of STAT3 in a wide variety of human tumors including hematological malignancies and solid tumors inducing cell proliferation, angiogenesis, invasion, and metastasis [123]. This cancer progression effect of STAT3 has been recognized in glioma cells in response to S100B/RAGE [124,125]. STAT3 is a transcription factor that regulates the expression of genes related to cell cycle, cell survival, and cancer malignancy. Once STAT3 is activated, it translocate to the nucleus where it promotes the translation of target genes associated with anti-apoptosis, angiogenesis, invasion, and migration [126]. Finally, we can control some of STAT3 cancer progression through inhibition of the S100B-RAGE pathway.

**Table 3:** Role of the S100 family-receptor for advanced glycation end product (RAGE) axis in cancer progression.

S100 Type	Cell Lines/Samples	Study Type	Mechanisms of Action	References
S100A4	Human osteosarcoma (II-11b)	In vitro	↑NF-***κ***B	[96]
	Human melanoma (A375)	In vitro	↑NF-***κ***B	[127]
	Human pancreatic cancer (BxPC-3)	In vitro	↑NF-***κ***B	[74]
	Human pancreatic carcinoma (MiaPACA-2)	In vitro	↑VEGF	[103]
	Human colorectal carcinoma (HCT116, SW620, and DLD-1)	In vitro	↑ERK	[95]
	Human melanoma (B16-F10)	In vitro	↑NF-***κ***B	[97]
	Human colorectal carcinoma (SW480 and LoVo)	In vitro	↑Akt, ↑mTOR, ↑p70S6K, ↑VEGF, ↓E-cadherin	[101]
	Thyroid cancer (human specimens)	In vitro	↑Cdc42, ↑ERK	[109]
	Human melanoma (A375)	In vitro	↓E-cadherin	[116]
	Human melanoma (A375)	In vitro	↑NF-***κ***B	[128]
S100A6	Nasopharyngeal carcinoma (human specimens)	In vivo	↑p38	[107]
S100A7	Human breast adenocarcinoma (MDA-MB-468)	In vitro	↑VEGF	[102]
	Aggressive triple-negative breast cancer (human specimens)	In vivo	↑ERK, ↑NF-***κ***B, ↑MMP9	[108]
	Human cervical cancer derived (C33A, HeLa, SiHa, and Caski)	In vitro	↑ERK	[129]
S100A8	Human prostate cancer (LNCaP and PC-3)	In vitro	↑NF-***κ***B, ↑p38, ↑ERK1/2	[105]
	Esophageal pre-neoplasia in the rat	In vivo	↑NF-***κ***B	[130]
	Colon carcinoma (MC38)	In vitro	↑NF-***κ***B, ↑ERK1/2, ↑SAPK/JNK	[106]
	Oral-esophageal tumor in mice	In vivo	↑NF-***κ***B	[114]
	Human breast cancer (MCF-7 and T47D)	In vitro	↑NF-***κ***B	[98]
	Hepatocellular carcinoma in mice	In vivo	↑ERK	[131]
	Squamous cell carcinoma (human specimens)	In vivo	↑p38, ↑SAPK/JNK, ↑ERK1/2	[132]
S100A9	Human prostate cancer (LNCaP and PC-3)	In vitro	↑NF-***κ***B, ↑p38, ↑ERK1/2	[105]
	Colon carcinoma (MC38)	In vitro	↑NF-***κ***B, ↑ERK1/2, ↑SAPK/JNK	[106]
	Human breast cancer (MCF-7 and T47D)	In vitro	↑NF-***κ***B	[98]
	Hepatocellular carcinoma in mice	In vivo	↑ERK	[131]
	Squamous cell carcinoma (human specimens)	In vivo	↑p38, ↑SAPK/JNK, ↑ERK1/2	[132]
	Human hepatocellular carcinoma (HepG2, SMMC-7721 and Huh7)	In vitro	↑p-p38, ↑p-ERK1/2	[133]
S100A14	Esophageal squamous cell carcinoma (KYSE180)	In vitro	↑ERK1/2, ↑NF-***κ***B	[115]
S100A16	Human prostate cancer (DU-145, LNCaP, and PC-3)	In vitro	↑Akt, ↑ERK, ↓p21, ↓p27	[121]
S100B	Human melanoma (WM115)	In vitro	↓p53	[119]
	Human large cell lung carcinoma (H1299)	In vitro	↓p53	[117]
	Human breast cancer (MCF-7)	In vitro	↓p53	[117]
	Human colorectal carcinoma (SW480)	In vitro	↑ERK1/2	[134]
	Human malignant melanoma (C8146A)	In vitro	↓p53	[118]
	Human neuroblastoma (SH-SY5Y)	In vitro	↑PI3K/Akt, ↑NF-***κ***B	[113]
	Human malignant melanoma (C8146A)	In vitro	↓p53	[135]
	Murine glioma (GL261)	In vitro	↑STAT3	[125]
	Ovarian cancer stem-like cell	In vitro	↓p53	[120]
	Glioma (C6)	In vitro	↑Akt1, ↑STAT3	[124]
S100P	Human pancreatic adenocarcinoma (BxPC-3 and MPanc-96)	In vitro	↑NF-***κ***B	[136]
	Human colon cancer (SW480)	In vitro	↑NF-***κ***B, ↑ERK1/2	[104]
	Human pancreatic cancer (BxPC-3)	In vitro	↑NF-***κ***B	[74]
	Human colorectal carcinoma (LS174T and SW480)	In vitro	↑miR-155	[137]
	Human pancreatic carcinoma (BxPC3)	In vitro	↑MMP9	[111]
	Human oral squamous cell carcinoma (OSCC)	In vitro	↑NF-***κ***B	[138]
	Human colorectal carcinoma (SW480 and LS174T)	In vitro	↑c-Fos, ↑AP-1, ↑miR-21	[139]
	Human nasopharyngeal carcinoma (C666-1)	In vitro	↑MMP2, ↑MMP9	[112]

**Akt**; protein kinase B, **AP-1**; activator protein 1, **Cdc42**; cell division control protein 42 homolog, **ERK**; extracellular signal-regulated kinase, **JNK**; c-Jun N-terminal kinase, **MMPs**; matrix metalloproteinases, **mTOR**; mammalian target of rapamycin, **NF-***κ***B**; nuclear factor kappa B, **p70S6K**; ribosomal protein S6 kinase B1, **SAPK**; stress-activated protein kinases, **STAT3**; signal transducer and activator of transcription 3, **VEGF**; vascular endothelial growth factor.The arrow pointing up (↑) means increase or upregulation. The arrow pointing down (↓) means decrease or downregulation.

## 5. RAGE-Inhibitors

RAGE signaling blocking in cell and animal models has shown that targeting RAGE affects the development and metastasis of cancer [140]. As discussed above, the pivotal role of RAGE in cancer progression caused by the induction of several cellular pathways is related to either cancer cell proliferation, migration, invasion, or metastasis. The goal of some studies was to discover new drugs able to alleviate or block the cancer progression. Still, these problems require further investigations. The respective studies are listed in Table 4 and discussed in the following paragraphs. 

### 5.1. Duloxetine

**Figure 2:**
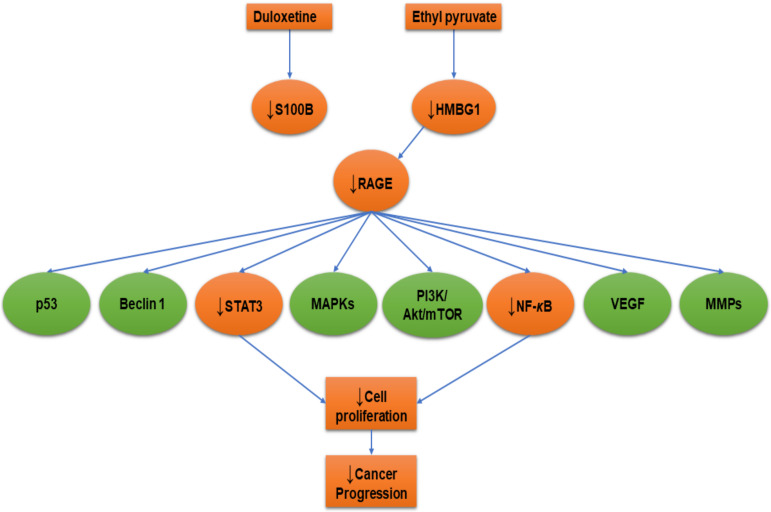
Receptor for advanced glycation end product (RAGE) inhibition with duloxetine and ethyl pyruvate. Orange colored shapes refer to the affected cellular molecules due to RAGE inhibition. The arrow pointing down (↓) means decrease or downregulation.

Cancer is often mixed with neuropathic pain. For this reason, frequently in patients with cancer-related neuropathic pain opioids such as anticonvulsants (e.g. pregabalin and gabapentin) and antidepressants (e.g. duloxetine and tricyclics) are used [141]. Duloxetine is a serotonin/noradrenaline reuptake inhibitor that is used as an antidepressant and is also prescribed for neuropathic pain [142] (Figure 2). There are a few studies that used duloxetine for chemotherapy-induced peripheral neuropathy [143]. Gao et al. [144] searched for S100B-inhibitors using a high-throughput screening cell-based S100B promoter-driven luciferase reporter assay. The authors treated mouse glioma cells (GL261) with duloxetine at a dose of 30 mg/kg by oral gavage for 14 days and determined that duloxetine inhibited S100B production and inhibited the growth of intracranial GL261 gliomas. These findings affirm the role of S100B in glioma progression. Thus, we think that it is important to develop more potent S100B-inhibitors for glioma therapy.

### 5.2. Ethyl Pyruvate

Ethyl pyruvate, the ethyl ester of pyruvic acid), has been shown to be an effective HMGB1-inhibitor in inflammation-related diseases and several cancers [145,146]. Pellegrini et al. [145] studied the effect of ethyl pyruvate on human malignant mesothelioma cells in tissue cultures and on tumor growth in vivo. The authors established that there was a significant impairment of HMGB1 secretion by malignant mesothelioma cells due to ethyl pyruvate, leading to reduction in RAGE expression and NF-***κ***B activation. Moreover, ethyl pyruvate reduced HMGB1 serum levels in mice and inhibited the growth of malignant mesothelioma xenografts. Therefore, ethyl pyruvate is an effective drug for malignant mesothelioma. In another in vitro study, ethyl pyruvate attenuated the non-small cell lung cancer cell lines growth, invasion, and migration and induced apoptosis via the downregulation of HMGB1-RAGE axis and the NF-***κ***B/STAT3 pathway [147] as illustrated in Figure 2. 

### 5.3. Hispidin

**Figure 3:**
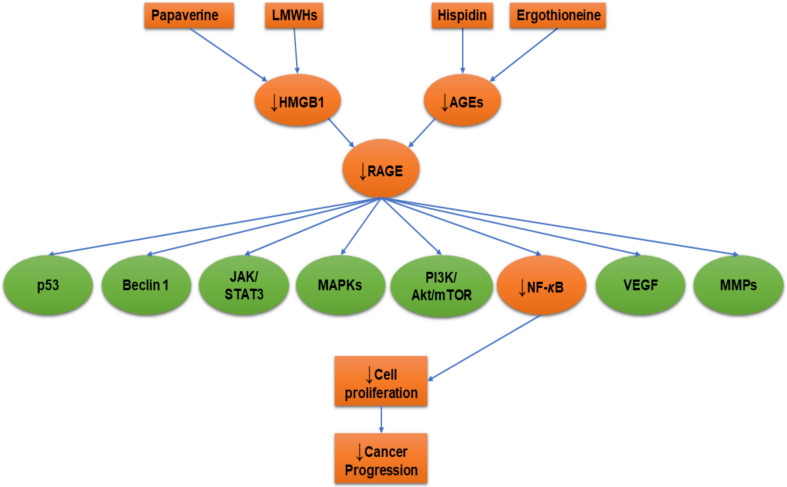
Receptor for advanced glycation end product (RAGE) inhibition with hispidin, ergothioneine, low-molecular-weight heparins (LMWHs), and papaverine. Orange colored shapes refer to the affected cellular molecules due to RAGE inhibition. The arrow pointing down (↓) means decrease or downregulation.

Natural plant products can be important for developing drug products. Hispidin is a polyphenol compound derived from *Phellinus linteus* and it has several biological activities such as antioxidant [148] and anticancer [149]. Hispidin could be a new gemcitabine chemosensitizer and potentially a synergistic agent to increase the gemcitabine therapeutic index to treat pancreatic cancer [150]. In addition, hispidin significantly induced apoptosis in colon cancer cells by generation of reactive oxygen species (ROS) [149]. Rat pheochromocytoma (PC12) cells were pre-incubated with 2μM of ergothioneine, thiol molecule synthesized by some fungi and bacteria, hispidin, or a combination of them. The results revealed a significant attenuation of AGEs’ formation, RAGE expression, and NF-***κ***B pathway activation through antioxidant activities [44]. Both the antioxidant compounds ergothioneine and hispidin counteracted the AGEs-RAGE axis-related induction of carcinogenesis (Figure 3).

### 5.4. Heparin 

The low-molecular-weight heparins (LMWHs) are an old class of anti-thrombotic drugs and tend to be the preferred anticoagulant in many indices that are important for modern hematology and oncology with patients who are at elevated risk of both hemorrhage and venous thromboembolism [151]. LMWH attenuated the HMGB1-induced NF-***κ***B activation through RAGE using an NF-***κ***B-dependent luciferase reporter assay and the HT1080 cell line. LMWH significantly inhibited the migration, invasion, tumor formation, and lung metastasis of HT1080^RAGE^ cells, but not of HT1080^mock^ or HT1080^dnRAGE^ cells [75] (Figure 3). The authors suggested that LMWH has therapeutic potential in patients with certain types of malignant tumors. In the same manner, chondroitin sulfate and heparan sulfate targeted RAGE and significantly decreased pulmonary metastasis [152].

### 5.5. Papaverine 

Papaverine, a non-narcotic opium alkaloid, is isolated from *Papaver somniferum*. Papaverine exhibited selective anticancer effects against several tumor cells [11,153]. An in vitro study was done to investigate the anti-RAGE effect of papaverine, optimized by the structure-based drug design system named conversion-to-small-molecules-through optimized-peptide strategy (COSMOS), in HT1080 human fibrosarcoma cells. Using RAGE- or dominant-negative RAGE-expressing HT1080 human fibrosarcoma cells, papaverine suppressed RAGE-dependent HT1080 human fibrosarcoma cell proliferation, migration, and invasion in a dose-dependent manner through a significant inhibition of RAGE-dependent NF-***κ***B driven by HMGB1 [11] (Figure 3). Furthermore, papaverine downregulated HMGB1 and RAGE along with significant inhibition of cell proliferation in human glioblastoma (U87MG and T98G) cell lines [153]. Therefore, papaverine could inhibit RAGE and is considered to be a promising anticancer drug.

**Table 4:** Receptor for advanced glycation end product (RAGE)-inhibitors that attenuated cancer progression.

Drug	Cell Line/Samples	Study Type	Mechanisms of Action	References
Chondroitin sulfate and heparan sulfate	Mice	In vivo	↓Lung metastasis	[152]
Duloxetine	Mouse glioma cells (GL261)/mice	In vitro/In vivo	↓S100B	[144]
Ergothioneine	Rat pheochromocytoma (PC12)	In vitro	↓AGEs, ↓RAGE, ↓NF-***κ***B	[44]
Ethyl pyruvate	Human malignant mesothelioma (MM)	In vitro	↓HMBG1, ↓RAGE, ↓NF-***κ***B	[145]
	Non-small cell lung cancer (A549, H520, and PC-9)	In vitro	↓HMGB1, ↓RAGE, ↓NF-***κ***B, ↓STAT3	[147]
Hispidin	Rat pheochromocytoma (PC12)	In vitro	↓AGEs, ↓RAGE, ↓NF-***κ***B	[44]
Heparin (low molecular weight)	Human fibrosarcoma (HT1080)	In vitro	↓NF-***κ***B	[75]
Papaverine	Human fibrosarcoma (HT1080)	In vitro	↓HMBG1, ↓RAGE, ↓NF-***κ***B	[11]
	Human glioblastoma (U87MG and T98G)		↓HMBG1, ↓RAGE	[153]

**AGEs**; advanced glycation end products**, NF-***κ***B**; nuclear factor kappa B, **STAT3**; signal transducer and activator of transcription 3 **RAGE;** receptor for advanced glycation end product.The arrow pointing up (↑) means increase or upregulation. The arrow pointing down (↓) means decrease or downregulation.

## 6. Conclusions

From the studies reviewed here, it can be concluded that RAGE-ligand complexes induce upregulation of an array of anti-apoptotic proteins and downregulate pro-apoptotic proteins to promote cancer cell progression, as illustrated in Figure 1. It is essential to screen for new anti-RAGE drugs with capabilities to control cancer progression. For further characterization of the effects of RAGE-ligands on cancer progression and for development of better treatments, we propose the following study points for consideration:

▪ Comparative studies of RAGE-ligands.
▪ The role of RAGE-ligands in cancer progression in primary cell culture of surgically removed tumor masses or cancer biopsies.
▪ The role of RAGE-ligands in cancer progression using cancer stem cells.
▪ The role of AGEs in colorectal cancer with therapeutic trials.
▪ Studies of the effect of RAGE-ligands’ pathway signaling on intrinsic pathway components such as cytochrome c, apoptotic protease activating factor 1 (Apaf-1), caspase-9, and caspase-3.
▪ Studies of the effect of RAGE-ligands’ pathway signaling on extrinsic pathway components such as tumor necrosis factor receptor-associated death domain (TRADD), Fas-associated death domain (FADD), caspase-8, and caspase-10.
▪ Studies of the effect of RAGE-ligands’ pathway signaling on Bcl-2 family, either the pro-apoptotic (BAX, BID, BAK, or BAD) or anti-apoptotic (Bcl-Xl and Bcl-2).
▪ Studies of the effect of RAGE-ligands’ pathway signaling on molecules that induce cell survival and metastasis including E-cadherin, hypoxia-inducible factor 1-alpha (HIF-1α), PTEN, and MDM2.
▪ Studies of the effect of RAGE-ligands’ pathway signaling on cyclin-dependent kinases (CDK-1, 2, 4, or 6) and regulatory cyclin subunits (cyclin A, B, Ds, or E).
▪ Studies of the effect of RAGE-ligands’ pathway signaling on molecules that facilitate cell survival and metastasis such as β-catenin, epidermal growth factor receptor (EGFR), VEGF, and vimentin.
▪ Discovery of new drugs that downregulate RAGE and its ligands to control cancer progression.
▪ The role of RAGE-ligands in cancer senescence and senotherapies.

## Abbreviations

Akt	protein kinase B
AP-1	activator protein 1
Apaf-1	apoptotic protease activating factor 1
ATP	adenosine triphosphate
Bad	Bcl-2-associated death promoter,
Bak	Bcl-2 homologous antagonist/killer
Bax	Bcl-2-associated X protein
Bcl-2;	B-cell lymphoma 2
Bcl-XL	B-cell lymphoma-extra-large
Cdc42	cell division control protein 42 homolog
CDK-1	cyclin-dependent kinase-1
CDK-2	cyclin-dependent kinase-2
CDK-4	cyclin-dependent kinase-4
CDK-6	cyclin-dependent kinase-6
ChREBP	carbohydrate responsive element binding protein
EGFR	epidermal growth factor receptor
ERK	extracellular signal-regulated kinase
FADD	Fas-associated death domain
HIF-1α	hypoxia-inducible factor 1-alpha
HMGB1	high-mobility group box1
JAK	Janus kinase
JNK	c-Jun N-terminal kinase
MAPK	mitogen-activated protein kinase
MDM2	mouse double minute 2 homolog
MKK6	mitogen-activated protein kinase kinase 6
MMPs	matrix metalloproteinases
mTOR	mammalian target of rapamycin,
Nrf-2	Nuclear factor (erythroid-derived 2)-like 2
NF-***κ***B	nuclear factor kappa B
p70S6K	ribosomal protein S6 kinase B1
PI3K	phosphatidylinositide 3-kinase
PTEN	phosphatase and tensin homolog
Rac1	Ras-related C3 botulinum toxin substrate 1
RAGE	receptor of advanced glycation end product
ROS	reactive oxygen species
SAPK	stress-activated protein kinases
STAT3	signal transducer and activator of transcription 3
TRADD	tumor necrosis factor receptor-associated death domain
VEGF	vascular endothelial growth factor
VEGF-C	vascular endothelial growth factor C.
